# Strengths and limitations of computer assisted telephone interviews (CATI) for nutrition data collection in rural Kenya

**DOI:** 10.1371/journal.pone.0210050

**Published:** 2019-01-30

**Authors:** Christine Lamanna, Kusum Hachhethu, Sabrina Chesterman, Gaurav Singhal, Beatrice Mwongela, Mary Ng’endo, Silvia Passeri, Arghanoon Farhikhtah, Suneetha Kadiyala, Jean-Martin Bauer, Todd S. Rosenstock

**Affiliations:** 1 World Agroforestry Centre, Nairobi, Kenya; 2 Vulnerability, Analysis, and Mapping Unit, United Nations World Food Programme, Rome, Italy; 3 Nutrition Division, United Nations World Food Programme, Rome, Italy; 4 Department of Population Health, London School of Hygiene and Tropical Medicine, London, United Kingdom; 5 Kenya Country Office, United Nations World Food Programme, Nairobi, Kenya; 6 United Nations World Food Programme, Brazzaville, Republic of the Congo; 7 CGIAR Research Program on Climate Change, Agriculture, and Food Security, Kinshasa, Democratic Republic of the Congo; 8 World Agroforestry Centre, Kinshasa, Democratic Republic of the Congo; University of Michigan, UNITED STATES

## Abstract

Despite progress in fighting undernutrition, Africa has the highest rates of undernutrition globally, exacerbated by drought and conflict. Mobile phones are emerging as a tool for rapid, cost effective data collection at scale in Africa, as mobile phone subscriptions and phone ownership increase at the highest rates globally. To assess the feasibility and biases of collecting nutrition data via computer assisted telephone interviews (CATI) to mobile phones, we measured Minimum Dietary Diversity for Women (MDD-W) and Minimum Acceptable Diet for Infants and Young Children (MAD) using a one-week test-retest study on 1,821 households in Kenya. Accuracy and bias were assessed by comparing individual scores and population prevalence of undernutrition collected via CATI with data collected via traditional face-to-face (F2F) surveys. We were able to reach 75% (n = 1366) of study participants via CATI. Women’s reported nutrition scores did not change with mode for MDD-W, but children’s nutrition scores were significantly higher when measured via CATI for both the dietary diversity (mean increase of 0.45 food groups, 95% confidence interval 0.34–0.56) and meal frequency (mean increase of 0.75 meals per day, 95% confidence interval 0.53–0.96) components of MAD. This resulted in a 17% higher inferred prevalence of adequate diets for infants and young children via CATI. Women without mobile-phone access were younger and had fewer assets than women with access, but only marginally lower dietary diversity, resulting in a small non-coverage bias of 1–7% due to exclusion of participants without mobile phones. Thus, collecting nutrition data from rural women in Africa with mobile phones may result in 0% (no change) to as much as 25% higher nutrition estimates than collecting that information in face-to-face interviews.

## Introduction

Undernutrition among mothers is one of the leading causes of neonatal death globally [1] and is responsible for the death of an estimated 3.5 million children under the age of five per year [2], as well as reduced educational attainment and economic productivity among adults [3]. Diet, and dietary diversity in particular, are closely linked with undernutrition among both women and young children [4], and has been identified as a key area for research [5] and programming [6] to combat malnutrition. In recognition of the global impact of malnutrition, the United Nations General Assembly declared a Decade of Action on Nutrition (2016–2026), and the Sustainable Development Goals have set the ambitious target of ending hunger and undernutrition by 2030 [7].

Advances in reducing undernutrition depend on the ability to monitor nutritional status of at-risk populations in order to respond rapidly to crises and scale-up successful programming. Both require systematic data collection at a high spatial resolution and temporal frequency [8,9]; however, current approaches used to collect nutrition data do not meet the requisite demand [10]. Nutrition data is typically obtained by physically visiting the study location and conducting face-to-face (F2F) household surveys at relatively high cost and with variable data quality [11, 12]. Consequently, nutrition data collection is limited in scope and frequency, and disproportionately rare in areas of insecurity, disease outbreak, or poor infrastructure [11, 13]. New approaches that can generate information at the right time and places are critical to scale effective and efficient programming and to meet global goals on undernutrition [7].

Mobile phones are increasingly used to conduct surveys at scale in low- and middle-income countries (LMICs) [14]. This is in part possible because subscriptions to mobile services are increasing rapidly, including in Sub-Saharan Africa where approximately 46% of the population was connected in 2015 [15]. Despite these gains in mobile penetration, many at risk and vulnerable populations (target beneficiaries) may be missed due to lack of connectivity. For example, poor, rural women remain the most under-represented group in terms of both mobile phone ownership and usage [16]. Regardless, the proliferation of mobile devices and data collection platforms has catalyzed their use in monitoring efforts in health, food security, household characterization, disaster response, and other fields [17–20].

Data collection on nutrition via mobile phones may present novel challenges compared with traditional F2F surveys. There are four primary methods of mobile-phone based data collection: short messaging service (SMS); interactive voice response (IVR) either via telephone keypad or speech recognition; unstructured supplementary service data (USSD); and computer-assisted telephone interviewing (CATI) either with a human or computer operator. Each mode has inherent strengths and limitations for use in the developing world [14, 21, 22]. While SMS is perhaps the most cost-effective data collection mode, it requires literate participants, preventing its widespread use, particularly in Africa [15]. IVR provides greater flexibility in the types of questions that can be asked and ability to reach low-literacy users compared to SMS and may have similar reliability to SMS in LMIC contexts [22, 23] (but see [24] for contrast). However, IVR with speech recognition can be challenging to implement in local languages due to the need to create word banks with many speakers [25], and generally, IVR suffers from both lower response and completion rates than other modes when used in survey contexts [14, 22, 26]. USSD requires close collaboration with telecom companies, which may not be available in all locations [27], and does not directly address monitoring or research questions. In contrast, CATI offers the flexibility to adapt questions and survey administration to local languages and phone usage patterns and does not require literate participants. In addition to mobile money applications, voice calling is the most common use of mobile phones in LMICs [28], making CATI a promising mode of mobile data collection for food security and nutrition applications.

Changes in data collection mode may introduce bias and thereby alter the measures and inferences generated from the data [29]. These biases mainly arise from two sources: sampling biases and the interaction between the enumerator and respondent. When surveys are conducted via mobile phones, those without access to mobile devices or networks are necessarily excluded from the survey, potentially creating a *non-coverage bias* in estimates of population level metrics. Although the digital divide is shrinking [30], cost is still a large barrier to mobile phone ownership in the developing world [28], and mobile phone ownership often correlates with wealth, particularly among poor or rural households [16, 31, 32]. As wealth is also generally correlated with better nutrition and food security [33, 34], this may bias estimates of population level nutrition indicators measured with mobile phones. Survey mode may also alter the *non-response bias* in the data, which results from differences in those who chose to participate in mobile phone surveys or not. For example, women tend to be less trusting of calls from unknown numbers than men [35], potentially limiting their participation in call-based surveys.

A second source of bias results from the interactions between the respondent and the survey administrator (or lack thereof). During F2F interviews enumerators are able to observe the local context of the respondent and possibly able to elicit accurate answers to survey questions via informed interrogation. Alternatively, the degree of sociality or anonymity of the interview process can also introduce bias in survey data [36]. In particular, respondents may be more reluctant to give responses that they find socially undesirable the more social the survey process is, due to physical or verbal presence of the survey administrator [37, 38]. Therefore, surveys conducted via mobile phones, including via CATI, may be less subject to social-desirability bias than those conducted via F2F interviews.

Here, we tested the accuracy and potential bias of using CATI to collect nutrition data through a test-retest survey on two nutrition indicators in households in rural Kenya. While Kenya has high rates of undernutrition, typical within sub-Saharan Africa (26% of children under five are stunted, and 11% are underweight [39]), it also has the highest rates of mobile penetration in East Africa (81 mobile subscriptions per 100 people compared to a regional average of 53 [40]) and the lowest gender gap in phone ownership (7% compared to 13% for Sub-Saharan Africa [35]), making Kenya an ideal country to evaluate the accuracy of data collection with mobile phones. We hypothesized that CATI would generate (a) individual dietary diversity scores equivalent to F2F, (b) equivalent population-level prevalence of dietary diversity to F2F, but (c) a non-coverage bias resulting in higher estimates of dietary diversity among phone users compared to non-phone users.

## Materials and methods

### Study sites

We selected Baringo and Kitui Counties in Kenya as study sites, as these counties differ in socioeconomic and environmental conditions, mobile phone access and network coverage (S1 Table). Baringo County (0° 28’ N, 35° 59’ E) is characterized by mixed crop-livestock farming systems in the highlands and pastoralism in the lowlands, and generally receives adequate rainfall for agriculture throughout the county [41]. In Baringo, approximately 52% of the population is below poverty line [42], 30% of children are stunted [43], and 50% of the population owns a mobile phone [16]. Kitui County (1° 22’ S, 38° 23’ E) is generally at lower elevation than Baringo and is dominated by agro-pastoralism with sorghum, millet, and small livestock. There is inadequate rainfall for agriculture in the easternmost parts of the county. Kitui has higher rates of poverty (60%), child stunting (46%), and lower mobile phone ownership (25%), than Baringo (see above references).

Study locations within each county were selected through a combination of purposeful and random sampling of administrative units. We included all districts within each county (Kitui has two districts and Baringo four). Within each district in Kitui, we purposefully selected the divisions with the highest and lowest number of households. From the two districts in Baringo with the highest population, we purposefully selected the divisions with the lowest number of households, while from the two districts with the lowest population, we selected the divisions with the highest number of households, for a total of four divisions in each county. Within each division, we randomly chose two sublocations, for a total of 32 sublocations representing the economic and geographic variation within each county.

### Indicators

We tested data collection mode with two internationally-validated nutrition indicators: Minimum Dietary Diversity for Women (MDD-W) [44] and Minimum Acceptable Diet (MAD) for infants and young children [45] (Table 1). MDD-W accesses the micronutrient adequacy in the diet of women of reproductive age, a critical predictor of both maternal and child nutrition. MAD assesses the adequacy of Infant and Young Child Feeding (IYCF) based on both dietary diversity (MDD) and meal frequency (MMF). We also included a sociodemographic indicator, the Kenya Progress Out of Poverty Index (PPI) [46] as a wealth proxy to assess differences in mode effect on MDD-W and MAD by wealth. The indicators can be collected in short surveys of approximately five minutes, do not rely on pictorial demonstrations of food groups, and are calculated on a scorecard methodology based on respondents’ answers to several questions. The indicators differ in target population, type of data generated in question response, and conversion of scores to population prevalence (Table 1). To meet the threshold for adequate dietary diversity, participants must have consumed at least five food groups out of ten in the past 24 hours for MDD-W and four out of seven for MDD. Recommended MMF is satisfied when either (a) breastfed infants less than nine months old eat at least twice a day, (b) breastfed infants older than nine months eat at least three times per day, or (c) non-breastfed infants regardless of age consume milk at least twice per day and other foods at least four times per day. Both MDD and MMF criteria must be satisfied for MAD. Finally, PPI raw scores are converted into below poverty-line (defined as $1.25/day) likelihoods using nonlinear conversion tables [46]. Thus, we were able to examine the equivalence between modes at three levels: population prevalence, individual indicator score, and responses to indicator subquestions.

**Table 1:** Summary of indicators.

Indicator	Measures	Target Population	No. Qs	Data Types	Score	Prevalence	Conversion to Prevalence
**MDD-W**: Minimum Dietary Diversity for Women	Micronutrient deficiency in women of reproductive age	Women aged 15–49 years	10	Binary	0–10	Proportion of women consuming at least 5 out of 10 food groups	Step function
**MAD**: Minimum Acceptable Diet	IYCF, children’s undernutrition	Caretakers of children aged 6–23.99 months	15	Binary Continuous	P/F	Proportion of children consuming a minimum acceptable diet	Compound step function
**MDD**: Minimum Dietary Diversity	IYCF, micronutrient deficiency, stunting	Caretakers of children aged 6–23.99 months	8	Binary	0–7	Proportion of IYC consuming at least 4 out of 7 food groups	Step function
**MMF**: Minimum Meal Frequency	IYCF, caloric deficiency, wasting	Caretakers of children aged 6–23.99 months	7	Continuous	0-∞	Proportion of IYC consuming an adequate number of meals	Conditional^1^ step function
**PPI**: Progress Out of Poverty Index	Below Poverty line	Adults	10	Binary Continuous Categorical	0–100	Likelihood of being below poverty line	Logistic or Exponential function^2^

Sector, target population, survey length, data type, and conversion of score to prevalence for indicators in this study.

^1^ MMF is considered a conditional step function because the inflection point of the function depends on the age and breastfeeding status of the child.

^2^ The type of function used to convert PPI score to poverty likelihood depends on the definition of poverty used (e.g. national poverty line, $1.25/day, etc.).

### Experimental design

**Fig 1:**
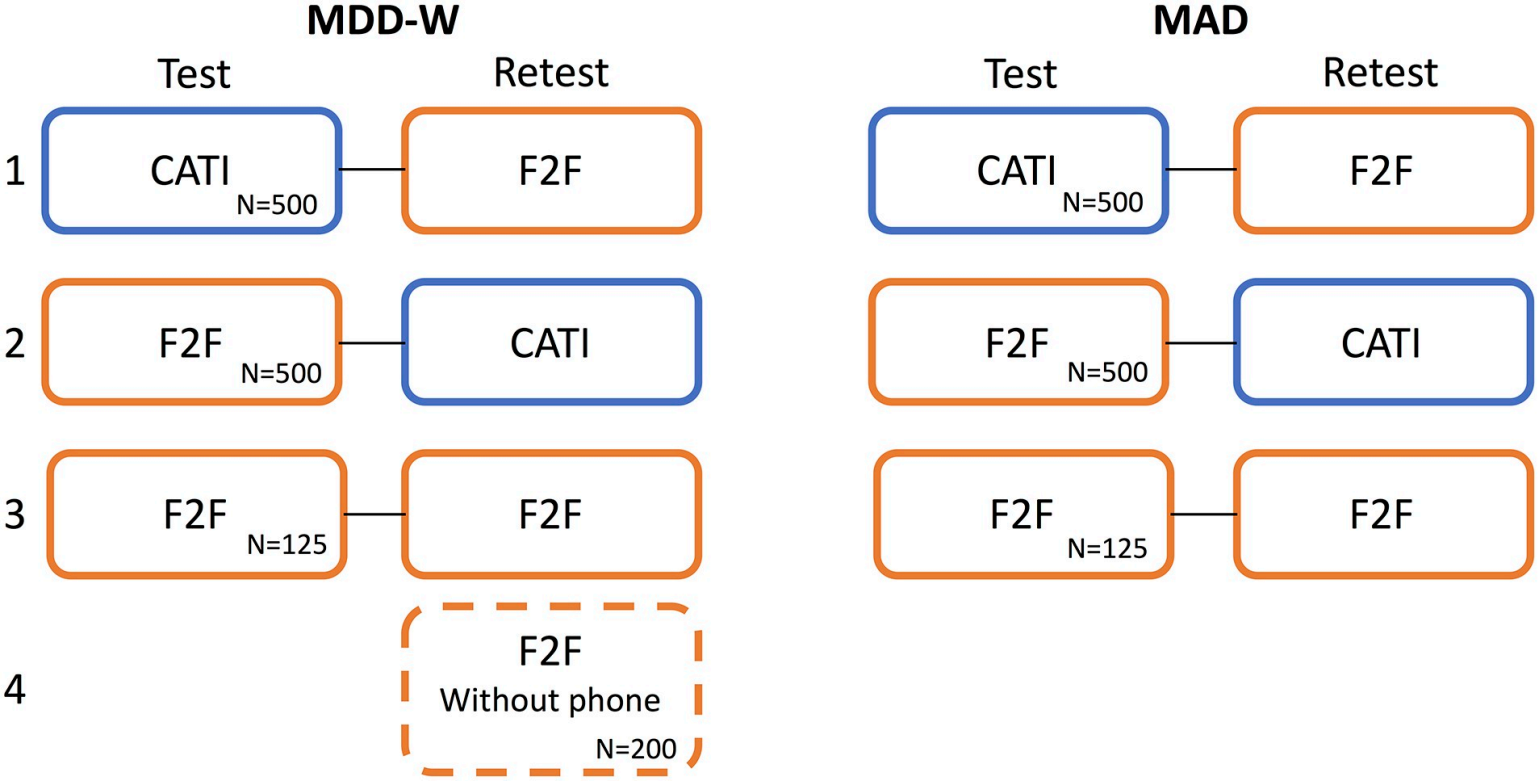
Experimental design. Test-retest mode experiment on two nutrition indicators, MDD-W and MAD. Survey consisted of four treatment arms: two main treatment arms testing for mode differences, arm 3 controlling for temporal effects and arm 4 controlling for non-coverage bias. Target sample sizes are indicated.

Two separate surveys were used concurrently for different target populations: MDD-W, PPI, and basic demography for women of reproductive age, and MAD, PPI and basic demography for adult caretakers of children aged 6–23.99 months. We used a test-retest design with four treatment arms (T1-T4) to evaluate the effect of data collection mode on nutrition indicators (Fig 1). In the main treatment arms T1 and T2, participants were interviewed with both CATI and F2F modes. We included two control arms to the experimental design. For the first control arm (T3), participants were interviewed via F2F mode in both rounds to understand potential learning and/or temporal effects. A fourth arm (T4) of F2F interviews for respondents with no phone access was included for MDD-W, to better understand non-coverage bias in conducting studies via CATI. Phone access was defined as owning a mobile phone or having access to one via intrahousehold sharing and was determined by asking potential participants.

Participants were systematically assigned to treatment arms during the test round, whereby participants meeting the inclusion criteria for the indicators were alternately assigned to T1 and T2, and every sixth house visited per day was assigned to T3. For participants in T1 (CATI first), phone numbers were collected and participants were called the next day. For participants in T2 and T3, F2F interviews were conducted immediately. In the retest round respondents were re-interviewed using the other mode, e.g. participants in T1 were relocated and interviewed F2F, while T2 participants were called via CATI. Participants in T3 were reinterviewed using F2F (Fig 1). The retest round occurred approximately nine days (8.7 ± 4.4 days) following the first survey, accounting for activities such as market days that may have altered diet choices when possible. G*Power [47] was used to calculate sample sizes to detect a mode bias of 1.5 in population prevalence using McNemar’s Exact Test for paired nominal data, assuming 25% of participants would switch indicator status (above or below threshold) between rounds. Although attrition rates in mobile surveys can vary dramatically with mode and location, for the sample size calculations we assumed a 15% attrition rate as CATI and F2F have low attrition rates, and studies with only two rounds have lower attrition rates than panel surveys [14]. For the power analyses, we used alpha = 0.2 and beta = 0.05 to minimize the rate of false equivalences. The resulting target sample size was 1000 participants, which we split evenly into the T1 and T2 arms. Control group sizes were chosen as 12.5% or 125 respondents for F2F controls, and 20% or 200 respondents for no-phone controls.

### Data collection

Thirty-two field enumerators (16 men and 16 women) and eight phone (3 men and 5 women) operators were recruited from local populations in Baringo and Kitui Counties to ensure familiarity with local languages, diets, and context. Enumerators and operators were trained together for two days to standardize survey methodology and interpretation of responses. To collect phone numbers and conduct F2F interviews, enumerator teams working simultaneously in Kitui and Baringo visited each identified sublocation in sequence. In each sublocation, enumerators sampled households semi-randomly, as enumerators traveled between households on foot, but were located in different parts of the sublocation, and did not sample adjacent households. Household sampling continued until 70 suitable households were identified for participation in the experiment. Only one participant was interviewed per household.

Both enumerators and operators used the same survey instrument, Enketo (Ona, Nairobi, Kenya and Washington, DC, USA), based on the Open Data Kit platform [48], to collect survey data. Operators used Enketo’s web form on desktops in United Nations World Food Programme’s in-house call center in Nairobi to conduct CATI interviews, while enumerators used a similar offline platform on tablets for the face-to-face surveys in the field. All data were uploaded daily to a centralized ODK server from where raw data was then extracted and analyzed.

### Data analysis

We use F2F results as the “control” scores and CATI scores as the “treatment”, as F2F is the standard mode of nutrition data collection. We evaluated effect of data collection mode in several ways. First, paired t-tests evaluated mean score as a function of mode for participants that received both CATI and F2F modes. At the population level, Kolmogorov-Smirnov tests compared distributional differences (mean, variance, skew and kurtosis) of indicator scores between modes. Equivalence Tests [49] against varying levels of difference assessed if differences by mode were clinically significant (large enough in magnitude to alter a nutritionist’s interpretation of the resultant data). Linear mixed-effects models were used to examine survey methodology effects on nutrition scores, such as mode bias, enumerator bias [50], or temporal effects. Using a top-down approach [51], we first used the most complex fixed effects of interest (a three-way interaction between survey mode, round and enumerator gender) and found the optimal structure for the random effects (such as county and enumerator). Then, using the resulting random effects, we determined the optimal fixed-effects structure. For model selection we computed both Akaike’s Information Criterion (AIC) and the Bayesian Information Criterion (BIC). Both AIC and BIC reward model explanatory power and penalize model complexity, but BIC also accounts for the number of observations in the dataset [51]. Where AIC and BIC disagreed on the best-fit model, we chose the model with the lowest AIC, in order to guard against false negatives (e.g. declaring there in no effect of survey mode when there may be). Differences in responses to indicator component questions at the individual level were examined via McNemar’s Exact Test and paired t-tests for categorical and continuous data respectively. Resulting p values were corrected for multiple testing using the false discovery rate method [52].

Non-coverage bias was assessed by comparing dietary diversity, PPI, and demographic data between the phone access and no phone access groups in the retest round for MDD-W. The magnitude of the non-coverage bias, or the relative change in population-level estimates of dietary diversity by only surveying women with mobile phones, was estimated as

$$Relative\;Bias=\frac{\frac{N_{2}}{N_{1}}({\overset{-}{Y}}_{1}-{\overset{-}{Y}}_{2})}{{\overset{-}{Y}}_{1}}$$

Where ${\overset{-}{Y}}_{1}$ and ${\overset{-}{Y}}_{2}$ are the mean MDD-W scores for women with and without phone access, respectively, and $\left(\frac{N_{2}}{N_{1}}\right)$ is the proportion of women without access to mobile phones [53]. We estimated mobile phone access in our population from published sources [16, 35]. All data analyses were conducted in R [54].

### Ethical considerations

The study protocol received research clearance and ethical approval from Kenya’s National Commission for Science, Technology and Innovation (NACOSTI), as well as the London School of Hygiene and Tropical Medicine (LSHTM). All methods were performed in accordance with NACOSTI and LSHTM guidelines. For all participants, oral informed consent was obtained by the enumerator and/or operator before beginning each survey. All efforts were made to ensure confidentiality of the participants. The data were stored in a password-protected computer and made accessible only to the core study team members. The analyses are presented in an aggregate format, phone numbers have been deleted, and all data has been anonymized. No incentives or remunerations were given for participation in this study.

## Results

### Survey completion

A total of 1,466 and 953 respondents for MDD-W and MAD, respectively, completed at least one round of the study (S2 Table). Twenty-four percent of participants did not participate in both survey rounds. Failure to reach participants twice was principally the result of unsuccessful phone interviews (65% of failures), rather than inability to relocate participants for F2F interviews (32% of failures). Inability to complete interviews via CATI, due to unanswered calls, poor network connectivity, unavailable interviewees, or wrong numbers was 20.1% across all indicators and locations (S2 Table). Subsequently, total sample sizes for MDD-W were 788 respondents in the main treatment arms (T1 & T2), 191 respondents in the F2F-F2F control arm (T3), and 210 in the no-phone control (T4). For MAD, 578 respondents were in the main treatment arms (T1 & T2) and 126 in the F2F-F2F control arm (T3).

### Prevalence of adequate diet with mode

Survey mode had inconsistent effects on measured prevalence of adequate diet in the sampled population (Table 2). Mode had no effect on prevalence of adequate dietary diversity in women of reproductive age (MDD-W) (Difference = 1.5% ± 2.2%, two-tailed Z = 0.692, p = 0.49). However, inferred prevalence of adequate dietary diversity for infants and young children (MDD) changed with mode, with 17.8% ± 2.6% higher prevalence of adequate dietary diversity measured via CATI (two-tailed Z = 6.738, p<0.00001). Similarly, the inferred prevalence of adequate meal frequency for infants and young children (MMF) was 12.3% ± 2.8% higher via CATI than F2F (two-tailed Z = 4.404, p = 0.00001). Similar to MDD and MMF, the compound metric MAD showed a difference in inferred prevalence with mode; it was 17.3% ± 2.3% higher via CATI than F2F (two-tailed Z = 7.399, p<0.00001). Overall, the rate of agreement in individual indicator status with mode was approximately 70%. For participants who received F2F interviews in both the test (R1) and retest (R2) rounds, there was no difference in the prevalence of adequate diet with round for women of reproductive age (MDD-W) or infants and young children (MAD), and the rate of agreement in individual indicator status between rounds was somewhat higher than between modes at 80% (S3 Table).

**Table 2:** Changes in nutrition indicators with data collection mode.

Indicator	N	F2F N (%)^a^	CATI N (%)^a^	Agreement N (%)^b^	X^2^^c^	p^c^
**MDD-W**	788	196 (24.9 ± 3.0)	208 (26.4 ± 3.2)	586 (74.4)	0.60	0.44
**MDD**	578	122 (21.1 ± 3.5)	225 (38.9 ± 4.0)	387 (67.0)	54.47	<0.0001
**MMF**	578	338 (58.5 ± 4.1)	409 (70.8 ± 3.8)	379 (65.5)	24.62	<0.0001
**MAD**	578	71 (12.3 ± 2.8)	171 (29.6 ± 3.9)	416 (72.0)	60.50	<0.0001

^a^ Number (N) and percentage (%) of respondents who met the threshold for adequate nutrition for the given indicator via each data mode. Confidence intervals around the percentage meeting the threshold were calculated using the Clopper-Pearson exact method.

^b^ Number (N) and percentage (%) of participants whose indicator status did not change between modes.

^c^ Based on McNemar’s Exact Test on respondents’ indicator status.

### Equivalence of scores with mode

**Fig 2:**
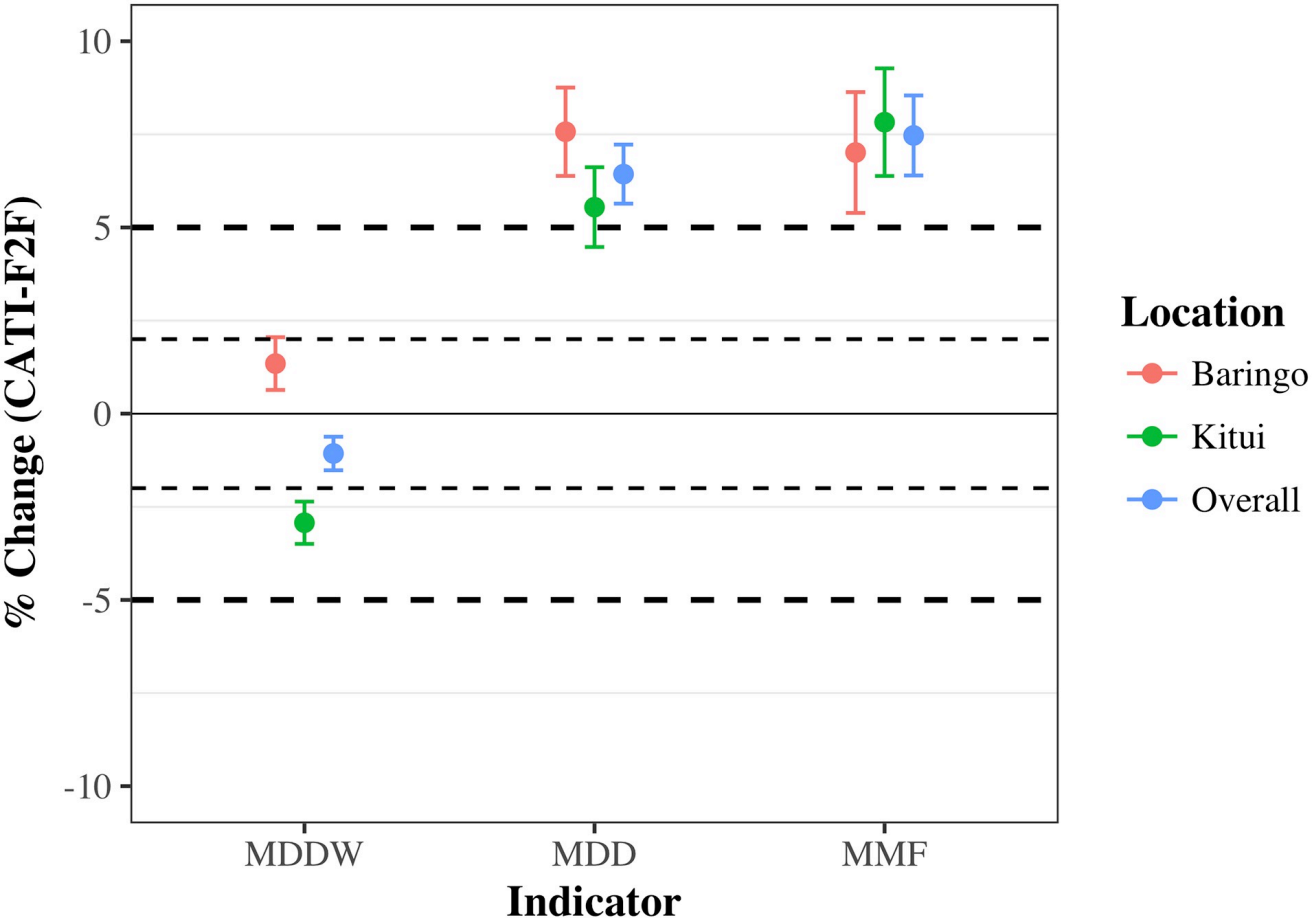
Equivalence of CATI and F2F scores for all nutrition indicators. While CATI and F2F give equivalent metric scores at the 2% for MDD-W, scores for MAD components MDD and MMF were only equivalent at the 9% level.

Paired responses revealed statistically significant effects of the CATI treatment across all indicators and locations. Both MDD-W and MAD scores were higher under CATI than F2F, with the exception of MDD-W in Kitui County, which was lower on average with CATI (Table 3). In addition to higher mean scores under CATI for the MDD and MMF components of MAD, we also find higher variance in MDD and MMF scores with the CATI mode (F = 0.68, p<0.0001), but no differences in the distribution of MDD-W scores with CATI. While differences in scores were numerically significant for all indicators and locations, the magnitude of the mode effect differed between MDD-W and MAD. For MDD-W, the mean difference between modes was 0.1 food groups (on a scale from 0–10). When converted into percentage differences, the modes give equivalent scores for MDD-W at the level of 2% difference, but yield non-equivalent scores at the 1% level (Fig 2). The magnitude of differences in score with mode was larger for the MAD components than with MDD-W. Overall, caregivers reported that children consumed an average of 0.45 more food groups per day (on a scale of 0–7 food groups) and ate an average of 0.75 more meals per day (average number of meals per day in this study was five, with 95% of scores falling at 10 or fewer meals per day) via CATI as compared to F2F; therefore, scores for MDD and MMF are only equivalent between modes at the 9% level (Fig 2).

**Table 3:** Changes in nutrition score with CATI by county.

Score	Kitui				Baringo			
	N	Difference	t	p	N	Difference	t	p
**MDD-W**	445	-0.29	5.20	<0.0001	343	0.14	1.97	0.049
**MDD**	357	0.39	5.24	<0.0001	251	0.53	6.36	<0.0001
**MMF**	357	0.76	5.32	<0.0001	251	0.72	4.45	<0.0001

Mean paired difference in respondent scores, CATI Score minus F2F Score. For MDD-W and MDD, units are food groups. Units for MMF are meals per day. All tests are two-tailed paired t-tests.

**Fig 3:**
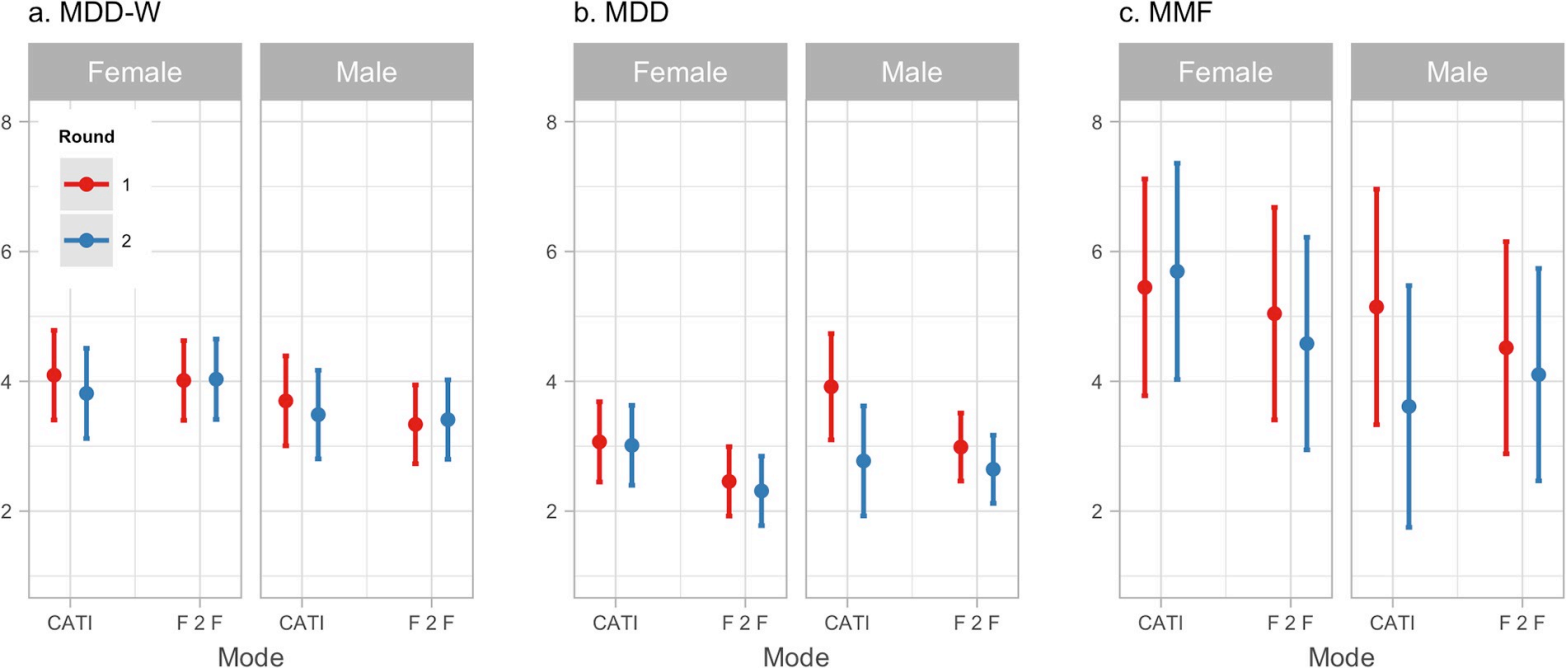
Interactions between survey mode, round and enumerator gender on nutrition indicator scores. Predicted nutrition indicator scores based on linear mixed-effects models for a) MDD-W in number of food groups, b) MAD MDD in number of food groups, and c) MAD MMF in number of meals per day. While only enumerator gender had an effect on MDD-W score, there were significant three-way interactions between enumerator gender, survey round and survey mode on MAD components MDD and MMF.

Scores for all nutrition indicators were lower in the retest round than in the test round (S3 Table). On average, MDD-W scores were reduced by 0.1 food groups (paired t = 3.10, p = 0.002), MDD by 0.22 food groups (paired t = 4.4, p<0.0001), and MMF by 0.2 meals per day (t = 2.18, p = 0.029). This effect was seen regardless of data collection mode. When accounting for differences in scores using linear mixed effects models, we found significant conditional effects of enumerators and locations across all three nutrition indicator scores (S4 and S5 Tables), which explained more than 50% of the variance in each indicator (S6 Table). Marginal effects of survey methodology explained a small but significant amount of residual variation in nutrition indicator scores, however, the influence of survey mode, round, and enumerator gender differed between indicators. For MDD-W, only enumerator gender had a significant impact on dietary diversity score, with males tending to give lower scores by 0.6 ± 0.1 (t = -3.95) food groups regardless of survey mode or round (S6 Table). For MAD components MDD and MMF, we found evidence for three-way interactions between survey mode, round, and enumerator gender (Fig 3). In particular, we found that male enumerators gave lower MDD and MMF scores in the retest survey round when conducting interviews using CATI by 0.9 ± 0.3 food groups (t = -3.03) and 1.8 ± 0.6 meals per day (t = -3.22), respectively.

### Food group mode effects

The mode of survey collection affected answers to 40% of questions related to specific food groups. Consumption of pulses was consistently reported more frequently via CATI than F2F across both dietary diversity indicators (MDD-W in Table 4 and MDD in Table 5). In contrast, reported dairy consumption was consistently higher in F2F interviews, except for consumption of milk by infants in Kitui County. The effect of mode on reporting consumption of locally uncommon food groups (those reported by less than 20% of the respondents, such as eggs, fruit, and vitamin-A rich foods, see S7 and S8 Tables) differed between the two indicators. In MDD-W, women tended to report more consumption of uncommon food groups under F2F (e.g. meat consumption is consistently higher via F2F). However, consumption of uncommon food groups was reported more frequently via CATI for the MDD module of MAD. Notably, caregivers reported significantly more consumption of fruits and vegetables by infants via CATI than F2F in both Kitui and Baringo Counties.

**Table 4:** Comparison of MDD-W food group reporting via CATI in Kitui and Baringo counties.

	Kitui (n = 445)				Baringo (n = 343)			
*Food Group*	Agreement (%)^a^	Δ N^b^	X^2^^c^	p^c^	Agreement (%)^a^	Δ N^b^	X^2^^c^	p^c^
*Grains*	98	-5	3.13	0.139	100	0	0	1.00
*Pulses*	76	+25	5.38	0.045	66	+46	18.1	<0.001
*Dairy*	67	-72	35.75	<0.001	74	-52	28.9	<0.001
*Dark Greens*	73	-15	1.65	0.257	80	+4	0.06	0.911
*Vegetables*	56	-4	0.12	0.821	51	+68	50.7	<0.001
*Fruits*	85	-14	2.56	0.165	79	-8	0.51	0.617
*Meat*	91	-21	11.61	0.003	89	-23	12.4	0.001
*Eggs*	98	0	0	1.00	91	-5	0.52	0.617
*Vitamin A*	90	-20	8.56	0.010	87	-8	1.07	0.540
*Nuts & Seeds*	100	0	NA	NA	98	-1	0	1

^a^ Percentage of respondents who reported the same consumption (either yes or no) of the food group under CATI and F2F.

^**b**^ The number of respondents who reported consuming that food group under CATI minus the number under F2F.

^**c**^ Based on McNemar’s Exact Test on respondents’ reporting under CATI vs. F2F and corrected for multiple testing with the false discovery rate method.

**Table 5:** Comparison of MDD food group reporting via CATI in Kitui and Baringo counties.

	Kitui (n = 357)				Baringo (n = 251)			
*Food Group*	Agreement (%)^a^	Δ N^b^	X^2^^c^	p^c^	Agreement (%)^a^	Δ N^b^	X^2^^c^	p^c^
*Grains*	90	-7	0.28	0.825	94	+4	2.4	0.330
*Pulses*	66	+22	4.01	0.083	62	+5	0.26	0.959
*Milk*	64	+37	12.34	0.001	87	-22	9.8	0.007
*Other Dairy*	98	0	0	1	99.6	+1	0	1
*Meat*	93	+2	0.042	0.924	94	-2	0.06	1
*Eggs*	94	+2	0.056	0.924	92	+2	0.05	1
*Vitamin A*	71	-10	0.52	0.739	64	+39	16.2	<0.0001
*Fruits & Vegs*	59	+73	41.2	<0.0001	48	+98	72.4	<0.0001
*Fortified Foods*	80	+37	19.34	<0.0001	94	+6	1.56	0.462
*Breastfed Ever*	97	+5	4.9	0.059	96	+2	0.9	0.623
*Breastfed Yesterday*	88	-21	7.23	0.019	90	-3	0	1

^a^ Percentage of respondents who reported the same consumption (either yes or no) of the food group under CATI and F2F.

^**b**^ The number of respondents who reported consuming that food group under CATI minus the number under F2F.

^**c**^ Based on McNemar’s Exact Test on respondents’ reporting under CATI vs. F2F and corrected for multiple testing with the false discovery rate method.

### Non-response bias

In both the MDD-W and MAD surveys, respondents who did not participate in both rounds were significantly younger and less likely to own a mobile phone than those who completed both rounds of the survey (S9 Table). They were also less likely to live in a household where the male head was formally employed; however, they did not differ in their likelihood of poverty as measured by PPI score, or in their education level. While non-respondents in the MDD-W survey did not differ from respondents in their MDD-W scores, in the MAD survey, non-respondents had lower MDD and MMF scores than those who completed both rounds of the survey.

### Non-coverage bias

Across all of the treatment arms, rounds and locations, dietary diversity and adequacy scores were correlated with measures of wealth such as the progress out of poverty (PPI) score (S10 and S11 Tables). PPI was also correlated with the number of phones owned by a household. When we look at participants in the T4 “no phone” control arm, we find that women without mobile phone access differed significantly from women with access (personal or household) in terms of their age, education level, and household characteristics (Table 6). Women without mobile phone access tended to be younger (-2 years, t = 2.73, p_cor_ = 0.025) and were more likely to have not completed any level of schooling (X^2^ = 5.89, p_cor_ = 0.039). They were also more likely to live in households without a male head (X^2^ = 15.23, p_cor_<0.0001), and more likely to fall below the poverty line (+10% more likely, t = -4.86, p_cor_<0.0001). Despite these differences, however, lack of phone access was not correlated with a difference in MDD-W score (t = 1.85, p_cor_ = 0.108). Given national mobile phone ownership rates for women in Kenya of 42.5% and weighting scores only based on phone ownership, the estimated relative bias for conducting nutrition surveys over mobile phones is 6.8% (overestimation of total population MDD-W score by 6.8% or 0.24 food groups). However, reported rates of phone ownership among women in our sample were much higher than the published national data for Kenya (S9 Table). If instead we calculate relative bias using the reported rate of phone ownership in our MDD-W survey of 85%, conducting nutrition surveys over mobile phones would only overestimate population level MDD-W by 0.9%.

**Table 6:** Differences between target women with and without access to a mobile phone.

	Phone Access	No Phone Access	t/X^2^	p_cor_
N	790	205		
*Age group*				
15–19	6% (4%-8%)	11% (7%-16%)	5.05	0.050
20–29	35% (32%-39%)	39% (32%-46%)	0.94	0.397
30–39	35% (32%-39%)	30% (24%-37%)	1.79	0.272
40–49	24% (21%-27%)	20% (15%-27%)	0.83	0.437
**Mean Age (years)**	**32.1 ± 8.6**	**30.2 ± 9.2**	**2.73**	**0.025**
*Education Level*				
**No school completed**	**11% (9%-14%)**	**18% (13%-24%)**	**5.89**	**0.039**
Completed primary	65% (62%-69%)	61% (54%-68%)	0.96	0.420
Completed secondary	17% (15%-20%)	18% (13%-24%)	0.03	0.910
Post-secondary	6% (4%-8%)	2% (0%-6%)	3.37	0.108
*Male Employment*				
**No male head**	**13% (11%-16%)**	**25% (19%-31%)**	**15.23**	**0.0006**
Not formally employed	62% (59%-66%)	64% (57%-70%)	0.12	0.819
Labor employment	9% (7%-11%)	4% (2%-8%)	5.08	0.050
**Non-labor employment**	**11% (9%-13%)**	**5% (2%-9%)**	**6.28**	**0.036**
Other	4% (3%-6%)	2% (0%-6%)	1.05	0.420
*Household Characteristics*				
**Household Size**	**6.6 ± 2.4**	**6.1 ± 2.2**	**2.93**	**0.018**
**PPI Score**	**36.9 ± 14.9**	**31.6 ± 13.7**	**4.79**	**<0.0001**
**Poverty Likelihood**	**48.2% ± 28.3%**	**58.5% ± 26.7%**	**-4.86**	**<0.0001**
*Nutrition Indicators*				
MDD-W	3.57 ± 1.24	3.39 ± 1.21	1.85	0.108

Data are proportions plus 95% confidence intervals unless otherwise noted. Differences were tested with X^2^ or t-tests for proportion or continuous data, respectively. All p values were corrected for multiple comparisons using false discovery rate methods.

## Discussion

In this study, we tested the utility of using computer assisted telephone interviewing (CATI) for cost effective, large scale nutrition data collection amongst women in rural Africa. We found that changing the data collection mode from traditional face-to-face (F2F) interviews to CATI resulted in higher nutrition indicator scores, but not for all indicators tested among rural women in Kenya. Dietary diversity scores did not differ in a clinically significant way (mean difference of less than 2%) with the mode of data collection when women were asked about their own diet, meaning that the inferenced population prevalence of adequate dietary diversity for women of reproductive age would have been the same regardless of the mode of data collection. In contrast, when women were asked about the diet of infants and young children in their care, the scores for MDD and MMF generated via CATI were 11–14% higher than those generated for the same child when the survey was administered F2F. This large difference in score between modes raised the inferred population prevalence of adequate dietary diversity (MDD, 18%), meal frequency (MMF, 12%), and overall diet (MAD, 17%) for infants and young children in our study populations. This mode effect also interacted with other survey methodology parameters, including survey round and the gender of the enumerator. Thus, we detected a significant, but inconsistent mode effect for nutrition data collection from rural women with mobile phones.

The observed difference in mode effect between MDD-W and MAD indicators could arise from several sources including: the types of data generated, the conversion between responses and indicator, the sensitivity of the indicator to the given local conditions, or the social perception of the questions asked. Both MDD-W and MAD ask participants to recall what they (or children within their care) ate in the previous 24 hours, and enumerators convert the responses into binary yes/no food group consumption scores, making it unlikely that differences in the types of data generated could lead to observed mode effects. Indeed, mode effects were observed for infant dietary diversity scores (MDD) but not for women’s dietary diversity scores (MDD-W). In contrast to MDD-W, MAD is a compound indicator that relies on meeting thresholds in two sub-indicators (MMF and MDD). MMF relies on participants giving a free, numeric response to questions on the frequency of consumption of milk, other milk products, semi-solid food and infant formula in the past 24 hours. Although, a mode effect in just one module of MAD would generate a mode effect in the compound indicator, we observed similar magnitude and direction of mode effects for both MDD and MMF components towards higher dietary diversity and more frequent meals reported via CATI. Therefore, it is unlikely that the observed differences in mode effect between MDD-W and MAD arose from differences in data type or conversion between score and prevalence.

Alternatively, the difference may be the consequence of the type of information asked with MAD. Questions that are considered sensitive, because they may reveal embarrassing or undesirable information about the respondent have been shown to show strong social-desirability bias, and the magnitude of this bias can change with survey mode [36–38]. In general, respondents tend to give more socially-desirable answers the more social the survey process is. For example, in Zimbabwe, youth were more likely to respond to sensitive questions about sexual health in both self-administered questionnaires with no interviewer and audio computer-assisted calls than in face-to-face interviews [55]. While social desirability may strongly affect self-reported responses in terms of dietary intake [56], asking a caregiver about the diet of infants in their care and their breastfeeding practices may be much more sensitive, and thus subject to stronger social-desirability bias than questions about their own eating habits or household assets. If infant care is more socially-sensitive than personal diet, this may explain why we see a mode effect of CATI with the MAD indicator, but not MDD-W.

Although we find evidence of a mode bias in potentially sensitive survey questions, the directionality of the bias is opposite to that predicted by social-desirability. We found that the less-social survey methodology of CATI actually resulted in higher nutrition indicator scores compared to F2F. While both F2F and CATI involve social interactions with an administrator in the same local language, we suspect that discomfort with receiving calls on mobile phones might have led to respondents giving more socially-desirable answers via CATI compared to F2F. Security concerns (including calls from unknown numbers, harassment, fraud, and spam) are the third largest barrier to women’s mobile phone use in Kenya after cost and network access [35]. Therefore, conducting nutrition interviews via CATI calls to mobile phones may have made the respondents less comfortable and thus more susceptible to the social-desirability bias than F2F interviews, resulting in the observed higher scores on MAD via CATI.

We also found evidence of enumerator gender effects on nutrition indicator scores. Male enumerators tended to record lower scores by more than half of a food group than female enumerators for MDD-W. For both components of MAD, enumerator gender interacted with survey mode and round. Men also tended to record lower nutrition indicator scores in the retest round of the survey when using CATI for MDD and MMF. Biases due to enumerator gender have been increasingly documented in the developing world [50] and are most common when survey questions deal with gender-sensitive topics [57]. MDD-W and MAD both rely on 24-hour recall methods and require enumerators to be familiar with the components of dishes and to probe respondents about aspects of their diet. Meal preparation is still largely the responsibility of women in Kenyan societies, which may mean that men are less familiar with the components of common dishes, and thus the food groups consumed. While gender biases in data recording have been well documented, how those biases interact with data collection mode and technology is less well known and deserves further study.

Collecting data via different survey modes can create sampling biases, when the reachable population via said mode differs from the general population (non-coverage bias), or when the participating population differs from the population that does not participate (non-response bias). A non-coverage bias is particularly important when data are intended to give information on aggregate level conditions. For example, in countries such as the United States and Australia, surveys conducted via landline telephones may generate substantial non-coverage bias as younger people are less likely to have landline telephones, skewing national estimates of demographic characteristics, health status [58], alcohol use [59], or election results [60]. In Sub-Saharan Africa, where mobile phone ownership is largely correlated with wealth and the majority of mobile phones are owned by men, conducting surveys via mobile phones may generate a non-coverage bias by disproportionately excluding the less wealthy and women. In this study, we also found that women of reproductive age who do not have personal or household mobile phones were significantly younger and less wealthy (as measured by PPI) than women who did have access to mobile phone. They also had marginally lower dietary diversity than women with mobile phone access, resulting in an estimated bias of 1–7% in MDD-W score from surveying only women with mobile phones. This magnitude of bias (0.03–0.25 food groups out of a mean score of 3.6 food groups) would not have changed the inferred population prevalence of adequately diverse diet among women of reproductive age. However, studies that intend to measure or monitor nutrition status with mobile calling among a broader population segment (e.g. including men and women, a larger age range, or both rural and urban populations) should weight survey results not only by the relative mode bias [53] but also by the demographics of the sampled population relative to the overall population [59]. While mobile phone surveys in rural Africa are likely to only select certain subpopulations, whether this bias will affect population level estimates of nutrition will depend on the proportion of the population that can be reached by mobile and the nutrition status of the population.

Even within the reachable population for a given mode, there may be differences between those who choose to participate in a survey, and those who choose not to. We found significant differences in mobile phone ownership, network access, and age between women who participated in both rounds of the nutrition survey and those who did not, either due to a missed or incomplete CATI call or a missed F2F interview. While these differences did not result in any differences in MDD-W among women of reproductive age, non-respondent caretakers of young children did report lower dietary diversity and lower meal frequency than those who participated in both rounds of the MAD survey. This likely reflects a nonrandom bias, whereby the likelihood of participating in the survey (via mobile phone ownership) is also correlated with the nutrition indicators of interest (higher infant nutrition indicators when measured by mobile interviews) [61]. This may explain why we see lower MAD scores but not lower MDD-W scores among non-responders, despite lower phone ownership and younger age for non-responders in both surveys.

Although we found substantial evidence for both a bias in data collected via CATI, a non-coverage bias among women who could not be reached via mobile phones, and a non-response bias among women who did not complete the survey, evidence of bias with survey mode does not necessarily invalidate the mode. Consistent bias may actually increase the utility of the mode [62], particularly when results can be calibrated (due to bias in score) or weighed to be representative (due to non-coverage bias) [53, 59]. We find that across indicators and locations, there was a consistent tendency for participants to report a better diet (higher dietary diversity, more frequent meals for infants and young children) via CATI. Although we only find marginal evidence of a non-coverage bias in terms of women’s dietary diversity, we did find that women without mobile phones in our study area had fewer assets, and that fewer assets was correlated with lower dietary adequacy. Based on our data, we estimate that conducting nutrition surveys via CATI could increase measured dietary scores by as little as 0% (no change) to as much as 25% (18% maximum mode effect + 7% maximum non-coverage bias), with concomitant effects on population prevalence of dietary adequacy. The exact magnitude of the bias will depend on the sensitivity of the survey questions to mode and the proportion and demographics of the population with access to mobile phones.

Despite the potential biases in using mobile phones to collect household survey data, there were numerous advantages to the mode. The cost of implementing the nutrition surveys was substantially lower using CATI compared to conducting traditional face to face surveys in the field. CATI costed US$ 5 per successful survey as compared to US$ 16 per successful survey via F2F. Furthermore, the CATI mode allowed us to reach participants despite an outbreak of insecurity in two of our sub-locations during the study. We also found high participation rates in the mobile phone administered surveys (approximately 75% participation), which is higher than mobile participation experienced in other CATI surveys in LMICs that do not provide phones to participants [14]. Early sensitization of communities in partnership with trusted local agencies, as well as multiple call attempts may have helped to increase participation in this study. Thus, mobile phones could be an important tool for monitoring vulnerable populations in places of high insecurity and geographical inaccessibility, as well as in situations where resources are limited.

In summary, our findings suggest that CATI can be used to successfully and cost-effectively collect MDD-W and MAD nutrition data among rural women in Kenya. Although dietary data collected via CATI is biased towards higher dietary diversity and more adequate diets for infants and young children as measured by MAD, this bias is consistent across locations and indicators, and may be corrected for if data collected via CATI must be compared to data collected via traditional F2F surveys. No mode effect of CATI was detected for women’s dietary diversity (MDD-W). Additionally, where mobile penetration rates are high, and the mobile accessible population does not differ from the population of interest, the non-coverage from excluding participants without mobile phones can be negligible. If nutrition data do not need to be benchmarked against F2F data and mobile penetration is high, then CATI can provide a particularly cost-effective method of collecting longitudinal nutrition data, even in rural communities and in areas of insecurity.

## Supporting information

S1 Table Study site characteristics. (DOCX)

S2 Table Survey success rates by mode and indicator. (DOCX)

S3 Table Changes in nutrition indicators with round. Changes in nutrition indicators with round for participants who received F2F interviews in both rounds (T3).
(DOCX)

S4 Table Mixed effects model selection. (DOCX)

S5 Table Mixed effects model variance components. (DOCX)

S6 Table Mixed effects model results. Fixed effect results from best-fit models of nutrition indicators as a function of survey mode, round, and enumerator gender.
(DOCX)

S7 Table Frequency of food group reporting. Frequency of food group reporting via CATI and F2F in Baringo and Kitui Counties for MDD-W.
(DOCX)

S8 Table Frequency of food group reporting. Frequency of food group reporting via CATI and F2F in Baringo and Kitui Counties for the MDD component of MAD.
(DOCX)

S9 Table Differences between responders and non-responders. Demographic, mobile access, and dietary adequacy differences between respondents who completed both rounds of the test-retest survey, and those who did not.
(DOCX)

S10 Table Correlations among MDD-W and demographic indicators. (DOCX)

S11 Table Correlations among MAD and demographic indicators. (DOCX)
