# Supplementary material for: Strengths and limitations of computer assisted telephone interviews (CATI) for nutrition data collection in rural Kenya
Source: PLoS One. 2019 Jan 30;14(1):e0210050. doi: 10.1371/journal.pone.0210050 (PMC6353544; doi:10.1371/journal.pone.0210050)
Supplement: S1 Table — (DOCX) [file pone.0210050.s001.docx]

**S1 Table**. **Study site characteristics.**

| **County** | **Pop (x1000)** | **Main Activities** | **Mean Annual Temp (C)^39^** | **Mean Annual Precip (mm)^39^** | **Poverty Rate (%)^40^** | **Child Stunting^41^** | **Child Wasting^41^** | **Main Diet** | **Mobile Phone Penetration^14^** | **Languages** |
| --- | --- | --- | --- | --- | --- | --- | --- | --- | --- | --- |
| Baringo | 556 | Pastoralism, bee-keeping, highland coffee & dairy | 16.0 (highlands)  22.8 (lowlands) | 1325 (highlands)  665 (lowlands) | 52.2% | 29.5 % | 6.9% | Maize meal, greens, milk | 50% | Kalenjin, Kiswahili |
| Kitui | 156 | Maize, sorghum and millet farming, agro-pastoralism | 20.0 (highlands)  24.9 (lowlands) | 925 (highlands)  550 (lowlands) | 60.4 % | 45.8% | 3.4% | Maize and beans | 25% | Kikamba, Kiswahili |
