# Supplementary material for: Strengths and limitations of computer assisted telephone interviews (CATI) for nutrition data collection in rural Kenya
Source: PLoS One. 2019 Jan 30;14(1):e0210050. doi: 10.1371/journal.pone.0210050 (PMC6353544; doi:10.1371/journal.pone.0210050)
Supplement: S2 Table — (DOCX) [file pone.0210050.s002.docx]

**S2 Table. Survey success rates by mode and indicator.**

|  |  | **MDD-W** | | | | |  | **MAD** | | | | |
| --- | --- | --- | --- | --- | --- | --- | --- | --- | --- | --- | --- | --- |
| **Arm** |  | **Test** | **Retest** | **N** | **%** | **Description** |  | **Test** | **Retest** | **N** | **%** | **Description** |
| **T1** |  | CATI | F2F | (503) |  |  |  | CATI | F2F | (400) |  |  |
|  |  | ✓ | ✓ | 347 | 69% | Successful |  | ✓ | ✓ | 283 | 71% | Successful |
|  |  | x | ✓ | 92 | 18% | Missed call |  | x | ✓ | 86 | 21% | Missed call |
|  |  | ✓ | x | 64 | 13% | Missed visit |  | ✓ | x | 31 | 8% | Missed visit |
|  |  |  |  |  |  |  |  |  |  |  |  |  |
| **T2** |  | F2F | CATI | (526) |  |  |  | F2F | CATI | (392) |  |  |
|  |  | ✓ | ✓ | 441 | 84% | Successful |  | ✓ | ✓ | 295 | 75% | Successful |
|  |  | ✓ | x | 83 | 16% | Missed call |  | ✓ | x | 83 | 21% | Missed call |
|  |  | x | ✓ | 2 | 0% | Missed visit |  | x | ✓ | 14 | 4% | Missed visit |
|  |  |  |  |  |  |  |  |  |  |  |  |  |
| **T3** |  | F2F | F2F | (227) |  |  |  | F2F | F2F | (161) |  |  |
|  |  | ✓ | ✓ | 191 | 84% | Successful |  | ✓ | ✓ | 126 | 78% | Successful |
|  |  | ✓ | x | 36 | 16% | Missed visit |  | ✓ | x | 35 | 22% | Missed visit |
|  |  |  |  |  |  |  |  |  |  |  |  |  |
| **T4** |  |  | F2F | (210) |  |  |  |  |  |  |  |  |
|  |  |  | ✓ | 210 | 100% | Successful |  |  |  |  |  |  |
