# Supplementary material for: Strengths and limitations of computer assisted telephone interviews (CATI) for nutrition data collection in rural Kenya
Source: PLoS One. 2019 Jan 30;14(1):e0210050. doi: 10.1371/journal.pone.0210050 (PMC6353544; doi:10.1371/journal.pone.0210050)
Supplement: S3 Table — Changes in nutrition indicators with round for participants who received F2F interviews in both rounds (T3). (DOCX) [file pone.0210050.s003.docx]

**S3 Table.** **Changes in nutrition indicators with round**.

| **Indicator** | **N** | **R1**  **N (%)^a^** | **R2**  **N (%)^a^** | **Agreement**  **N (%)^b^** | **X^2c^** | **p^c^** |
| --- | --- | --- | --- | --- | --- | --- |
| **MDD-W** | 189 | 45 (23.8) | 39 (20.6) | 147 (77.8) | 0.595 | 0.44 |
| **MDD** | 126 | 27 (21.4) | 24 (19.0) | 97 (77.0) | 0.14 | 0.71 |
| **MMF** | 126 | 72 (57.1) | 75 (59.5) | 101 (80.2) | 0.11 | 0.74 |
| **MAD** | 126 | 16 (12.7) | 15 (11.9) | 107 (84.9) | 0 | 1 |

Changes in nutrition indicators with round for participants who received F2F interviews in both rounds (T3).

^a^ The number (N) and percentage (%) of respondents who met the threshold for the given indicator in each round.

^b^ The number (N) and percentage (%) of participants whose indicator status did not change between rounds.

^c^ X^2^ and p are from McNemar’s Exact Test on respondents’ indicator status.
