# Supplementary material for: Strengths and limitations of computer assisted telephone interviews (CATI) for nutrition data collection in rural Kenya
Source: PLoS One. 2019 Jan 30;14(1):e0210050. doi: 10.1371/journal.pone.0210050 (PMC6353544; doi:10.1371/journal.pone.0210050)
Supplement: S4 Table — (DOCX) [file pone.0210050.s004.docx]

**S4 Table. Mixed effects model selection.**

|  | **Indicator** | **MDD-W** | | **MDD** | | **MMF** | |
| --- | --- | --- | --- | --- | --- | --- | --- |
|  |  | **AIC** | **BIC** | **AIC** | **BIC** | **AIC** | **BIC** |
| *Random Effects Structure* | |  |  |  |  |  |  |
| m1 | ~Mode x Round x Gender (=FE) | 5227 | 5275 | 3678 | 3723 | 5186 | 5232 |
| m2 | ~FE + (1\|Individual) | 5009 | 5063 | 3595 | 3644 | 5112 | 5162 |
| m3 | ~FE + (1\|County) | 5096 | 5150 | 3636 | 3687 | 5046 | 5096 |
| m4 | ~FE + (1\|Enumerator) | 5019 | 5073 | 3564 | 3614 | 5021 | 5071 |
| m5 | ~FE + (1\|County/Enumerator) | 5018 | 5078 | 3565 | 3620 | 5009 | 5065 |
| m6 | ~FE **+ (1\|Individual) + (1\|County/Enumerator)** | **4838** | **4903** | **3487** | **3548** | **4960** | **5021** |
|  |  |  |  |  |  |  |  |
| *Fixed Effects Structure* | |  |  |  |  |  |  |
| m6 | **~Mode x Round x Gender + (RE)** | 4838 | 4903 | **3487** | 3547 | **4960** | 5021 |
| m7 | ~Mode x Round + Mode x Gender + Round x Gender + (RE) | 4835 | 4894 | 3493 | 3549 | 4969 | 5024 |
| m8 | ~Mode x Round + Mode x Gender + (RE) | 4830 | 4885 | 3503 | 3553 | 4971 | 5022 |
| m9 | ~Mode x Round + Round x Gender + (RE) | 4834 | 4887 | 3491 | 3542 | 4968 | 5019 |
| m10 | ~Mode x Gender + Round x Gender + (RE) | 4835 | 4889 | 3490 | 3541 | 4967 | 5017 |
| m11 | ~Mode x Round + Gender + (RE) | 4829 | 4878 | 3501 | 3547 | 4971 | 5016 |
| m12 | ~Mode x Gender + Round + (RE) | 4831 | 4879 | 3500 | 3545 | 4970 | 5016 |
| m13 | ~Round x Gender + Mode + (RE) | 4834 | 4882 | 3489 | 3534 | 4966 | 5012 |
| m14 | ~Mode + Round + Gender + (RE) | 4829 | 4872 | 3497 | 3538 | 4970 | 5010 |
| m15 | ~Mode + Round + (RE) | 4837 | 4875 | 3498 | 3533 | 4972 | 5007 |
| m16 | ~Mode + Gender + (RE) | 4827 | 4865 | 3512 | 3547 | 4971 | 5006 |
| m17 | ~Round + Gender + (RE) | 4826 | 4863 | 3502 | 3537 | 4971 | 5006 |
| m18 | **~Round + (RE)** | 4834 | 4866 | 3500 | **3530** | 4975 | 5005 |
| m19 | **~Gender + (RE)** | **4824** | **4856** | 3516 | 3547 | 4972 | **5002** |
| m20 | **~Mode + (RE)** | 4836 | 4868 | 3513 | 3543 | 4972 | **5003** |

Table of model fit parameters for linear mixed effects models explaining variation in scores for MDD-W and MAD components MDD and MMF. We first fit the optimal random effects structure using a complex fixed effects structure (FE, shown in model m1), and then fit the fixed effects using the optimal random effects structure (RE, shown in model m6). Lowest criterion values for each model and step are shown in bold. Models were fit using the restricted maximum likelihood estimates (R package lme4). R code is available upon request.
