# Supplementary material for: Strengths and limitations of computer assisted telephone interviews (CATI) for nutrition data collection in rural Kenya
Source: PLoS One. 2019 Jan 30;14(1):e0210050. doi: 10.1371/journal.pone.0210050 (PMC6353544; doi:10.1371/journal.pone.0210050)
Supplement: S5 Table — (DOCX) [file pone.0210050.s005.docx]

**S5 Table. Mixed effects model variance components.**

|  | **MDD-W** | | **MDD** | | **MMF** | |
| --- | --- | --- | --- | --- | --- | --- |
| **Random Effect** | **LRT** | **p** | **LRT** | **p** | **LRT** | **p** |
| (1\|County) | 6.4 | 0.0112 | 1.4 | 0.242 | 14.7 | 0.0001 |
| (1\|Enumerator/County) | 81.1 | <0.0001 | 76.7 | <0.0001 | 43.0 | <0.0001 |
| (1\|HHID) | 181.1 | <0.0001 | 79.7 | <0.0001 | 51.1 | <0.0001 |

Likelihood ratio tests (LRT) and corresponding p values for variance components of best-fit linear mixed effects models presented in Table S4. Likelihood ratio tests were computed using R package lmerTest.
