# Supplementary material for: Strengths and limitations of computer assisted telephone interviews (CATI) for nutrition data collection in rural Kenya
Source: PLoS One. 2019 Jan 30;14(1):e0210050. doi: 10.1371/journal.pone.0210050 (PMC6353544; doi:10.1371/journal.pone.0210050)
Supplement: S6 Table — Fixed effect results from best-fit models of nutrition indicators as a function of survey mode, round, and enumerator gender. (DOCX) [file pone.0210050.s006.docx]

**S6 Table. Mixed effects model results.**

|  |  | **MDDW** | |  | **MDD** | |  | **MMF** | |
| --- | --- | --- | --- | --- | --- | --- | --- | --- | --- |
| **Model Fit** |  | *R^2^m = 0.05, R^2^c = 0.58* | |  | *R^2^m = 0.09, R^2^c = 0.54* | |  | *R^2^m = 0.06, R^2^c = 0.52* | |
| **Fixed Effect** |  | **Estimate** | **t** |  | **Estimate** | **t** |  | **Estimate** | **t** |
| Intercept |  | **4.00 ± 0.31** | **12.91** |  | **3.07 ± 0.31** | **9.72** |  | **5.45 ± 0.85** | **6.39** |
| Mode (CATI) |  |  |  |  | **0.61 ± 0.31** | **1.97** |  | 0.41 ± 0.40 | 1.02 |
| Round (Retest) |  |  |  |  | -0.05 ± 0.10 | -0.53 |  | 0.25 ± 0.19 | 1.30 |
| Gender (Male) |  | **-0.58 ± 0.1** | **-3.95** |  | **0.84 ± 0.44** | **1.92** |  | -0.30 ± 0.56 | -0.54 |
| Mode x Round |  |  |  |  | 0.09 ± 0.18 | 0.53 |  | **0.71 ± 0.33** | **2.14** |
| Mode x Gender |  |  |  |  | 0.33 ± 0.50 | 0.64 |  | 0.23 ± 0.65 | 0.34 |
| Round x Gender |  |  |  |  | **-1.09 ± 0.23** | **-4.68** |  | **-1.78 ± 0.45** | **-3.95** |
| Md x Rnd x Gen |  |  |  |  | **-0.89 ± 0.29** | **-3.03** |  | **-1.83 ± 0.57** | **-3.22** |

Table of fixed-effects values from best-fit mixed effects models of nutrition indicator scores (S4 Table), showing estimates, standard errors, and t values. Units for MDDW and MDD are in food groups, while MMF is number of meals. Marginal and conditional R^2^ values, estimated with R package MuMIn, are shown for each model. The significance of coefficients at the 95% confidence level, shown in bold, was determined by bootstrapping with R package lmeresampler, using 1000 iterations. R code is available upon request.
