# Supplementary material for: Strengths and limitations of computer assisted telephone interviews (CATI) for nutrition data collection in rural Kenya
Source: PLoS One. 2019 Jan 30;14(1):e0210050. doi: 10.1371/journal.pone.0210050 (PMC6353544; doi:10.1371/journal.pone.0210050)
Supplement: S7 Table — Frequency of food group reporting via CATI and F2F in Baringo and Kitui Counties for MDD-W. (DOCX) [file pone.0210050.s007.docx]

**S7 Table**. **Frequency of MDD-W food group reporting.**

|  |  | **Kitui (n=445)** | | | |  | **Baringo (n=343)** | | | |
| --- | --- | --- | --- | --- | --- | --- | --- | --- | --- | --- |
| ***Food Group*** |  | **F2F**  **(N)** | **CATI (N)** | **∆ N** | **∆ Rank** |  | **F2F**  **(N)** | **CATI (N)** | **∆ N** | **∆ Rank** |
| *Grains* |  | 444 | 439 | -5 | 0 |  | 343 | 343 | 0 | 0 |
| *Pulses* |  | 347 | 372 | +25 | 0 |  | 195 | 241 | +46 | +1 |
| *Dairy* |  | 252 | 180 | -72 | -1 |  | 289 | 237 | -52 | -2 |
| *Dark Greens* |  | 104 | 80 | -14 | 0 |  | 279 | 283 | +4 | +1 |
| *Vegetables* |  | 249 | 244 | -5 | +1 |  | 141 | 235 | +68 | 0 |
| *Fruits* |  | 45 | 31 | -14 | 0 |  | 58 | 50 | -8 | 0 |
| *Meat* |  | 40 | 19 | -21 | 0 |  | 32 | 9 | -23 | -2 |
| *Eggs* |  | 5 | 5 | 0 | 0 |  | 19 | 14 | -5 | +1 |
| *Vitamin A* |  | 32 | 12 | -20 | 0 |  | 27 | 19 | -8 | +1 |
| *Nuts & Seeds* |  | 0 | 0 | 0 | 0 |  | 4 | 3 | -1 | 0 |

Frequency of food group reporting via CATI and F2F in Baringo and Kitui Counties for MDD-W.
