# Supplementary material for: Strengths and limitations of computer assisted telephone interviews (CATI) for nutrition data collection in rural Kenya
Source: PLoS One. 2019 Jan 30;14(1):e0210050. doi: 10.1371/journal.pone.0210050 (PMC6353544; doi:10.1371/journal.pone.0210050)
Supplement: S8 Table — Frequency of food group reporting via CATI and F2F in Baringo and Kitui Counties for the MDD component of MAD. (DOCX) [file pone.0210050.s008.docx]

**S8 Table**. **Frequency of MAD food group reporting.**

|  |  | **Kitui (n=357)** | | | |  | **Baringo (n=251)** | | | |
| --- | --- | --- | --- | --- | --- | --- | --- | --- | --- | --- |
| ***Food Group*** |  | **F2F**  **(N)** | **CATI (N)** | **∆ N** | **∆ Rank** |  | **F2F**  **(N)** | **CATI (N)** | **∆ N** | **∆ Rank** |
| *Grains* |  | 308 | 301 | -7 | 0 |  | 241 | 245 | +4 | 0 |
| *Fruits & Vegs* |  | 150 | 223 | +73 | 0 |  | 56 | 154 | +98 | +2 |
| *Milk* |  | 119 | 156 | +37 | 0 |  | 238 | 216 | -22 | 0 |
| *Vitamin A* |  | 99 | 89 | -10 | -1 |  | 110 | 149 | +39 | -1 |
| *Pulses* |  | 76 | 98 | +22 | +1 |  | 80 | 85 | +5 | -1 |
| *Fortified Foods* |  | 31 | 68 | +37 | 0 |  | 6 | 12 | +6 | +1 |
| *Meat* |  | 16 | 18 | +2 | 0 |  | 12 | 10 | -2 | -2 |
| *Eggs* |  | 10 | 12 | +2 | 0 |  | 11 | 13 | +2 | +1 |
| *Other Dairy* |  | 3 | 3 | 0 | 0 |  | 0 | 1 | +1 | 0 |
| *Breastfed Ever* |  | 319 | 324 | +5 | NA |  | 247 | 249 | +2 | NA |
| *Breastfed Yesterday* |  | 276 | 255 | -21 | NA |  | 200 | 197 | -3 | NA |

Frequency of food group reporting via CATI and F2F in Baringo and Kitui Counties for the MDD component of MAD.
