# Supplementary material for: Strengths and limitations of computer assisted telephone interviews (CATI) for nutrition data collection in rural Kenya
Source: PLoS One. 2019 Jan 30;14(1):e0210050. doi: 10.1371/journal.pone.0210050 (PMC6353544; doi:10.1371/journal.pone.0210050)
Supplement: S9 Table — Demographic, mobile access, and dietary adequacy differences between respondents who completed both rounds of the test-retest survey, and those who did not. (DOCX) [file pone.0210050.s009.docx]

**S9 Table: Comparison of Responders and Nonresponders.**

|  | **MDD-W** | | **MAD** | |
| --- | --- | --- | --- | --- |
|  | **Responders** | **Nonresponders** | **Responders** | **Nonresponders** |
| N | 983 | 286 | 711 | 262 |
|  |  |  |  |  |
| *Age group* |  |  |  |  |
| 19 or younger^a^ | **7% (6%-9%)*** | **13% (10%-18%)*** | 7% (6%-9%) | 9% (6%-13%) |
| 20-29 | 34% (31%-37%) | 43% (37%-49%) | 52% (48%-55%) | 57% (51%-63%) |
| 30-39 | 35% (32%-38%)**^.^** | 27% (23%-33%)**^.^** | 31% (28%-35%) | 27% (22%-33%) |
| 40-49 | **24% (22%-27%)*** | **16% (12%-21%)*** | 7% (5%-9%) | 5% (3%-8%) |
| 50 or older |  |  | 3% (2%-4%) | 2% (0%-4%) |
| Mean Age (years) | **32.1 ± 8.8***** | **29.0 ± 8.8***** | **29.1 ± 8.3*** | **27.7 ± 7.5*** |
|  |  |  |  |  |
| *Education Level* |  |  |  |  |
| No school completed | 12% (10%-15%) | 9% (6%-13%) | 12% (9%-14%) | 12% (8%-16%) |
| Completed primary | 63% (59%-66%) | 63% (57%-69%) | 60% (56%-64%) | 65% (59%-71%) |
| Completed secondary | 19% (17%-22%) | 22% (17%-27%) | 21% (18%-24%) | 15% (11%-20%) |
| Post-secondary | 6% (5%-8%) | 6% (3%-9%) | 7% (5%-9%) | 8% (5%-12%) |
|  |  |  |  |  |
| *Male Employment* |  |  |  |  |
| No male head | 14% (12%-17%) | 15% (11%-20%) | 9% (7%-11%) | 11% (8%-16%) |
| Not formally employed | **54% (50%-57%)*** | **63% (57%-69%)*** | 52% (49%-56%)**^.^** | 61% (54%-67%)**^.^** |
| Labor employment | 10% (8%-12%) | 7% (5%-11%) | **12% (9%-14%)*** | **5% (3%-8%)*** |
| Non-labor employment | 12% (10%-15%) | 9% (6%-13%) | 13% (11%-16%) | 12% (9%-17%) |
| Other | **10% (8%-12%)*** | **5% (3%-8%)*** | 14% (11%-16%) | 11% (8%-16%) |
|  |  |  |  |  |
| *Household Characteristics* |  |  |  |  |
| Household Size (persons) | 6.5 ± 2.4 | 6.5 ± 2.7 | 6.3 ± 2.3 | 6.3 ± 2.4 |
| PPI Score | 37.9 ± 15.4 | 36.4 ± 15.0 | 40.8 ± 16.2 | 38.8 ± 16.0 |
| Poverty Likelihood | 46.8% ± 29.3% | 50.1% ± 28.9% | 41.4% ± 30.4% | 45.8% ± 28.7% |
| Number Phones in HH | **1.9 ± 1.1**** | **1.7 ± 0.9**** | 1.7 ± 1.0**^.^** | 1.6 ± 1.0**^.^** |
| Phone Ownership | **88% (86%-90%)*** | **80% (75%-84%)*** | **86% (83%-88%)***** | **74% (69%-80%)***** |
|  |  |  |  |  |
| *Nutrition Indicators* |  |  |  |  |
| MDD-W (food groups) | 3.36 ± 1.34 | 3.74 ± 1.29 |  |  |
| MDD (food groups) |  |  | **2.90 ± 1.28***** | **2.53 ± 1.21***** |
| Number of meals per day |  |  | **4.95 ± 2.29***** | **4.28 ± 2.17***** |

Data are proportions plus 95% confidence intervals unless otherwise noted. Differences were tested with X^2^ or t-tests for proportion or continuous data, respectively. All p values were corrected for multiple comparisons using false discovery rate methods. ^.^ p<0.1, * p<0.05, ** p<0.005, *** p<0.0005

^a^For MDD-W, respondents were women of reproductive age (15-49 years old inclusive), whereas for MAD respondents were the primary caretakers of infants aged 6-23 months, and could be of any age.
