# Supplementary material for: Strengths and limitations of computer assisted telephone interviews (CATI) for nutrition data collection in rural Kenya
Source: PLoS One. 2019 Jan 30;14(1):e0210050. doi: 10.1371/journal.pone.0210050 (PMC6353544; doi:10.1371/journal.pone.0210050)
Supplement: S10 Table — (DOCX) [file pone.0210050.s010.docx]

**S10 Table.** **Correlations among MDD-W and demographic indicators.**

|  | **Age** | **HH Size** | **Phones** | **PPI** | **MDD-W** |
| --- | --- | --- | --- | --- | --- |
| **Age** | 1.000 |  |  |  |  |
| **HH Size** | 0.194 | 1.000 |  |  |  |
| **Phones** | 0.077 | 0.167 | 1.000 |  |  |
| **PPI** | -0.066 | -0.468 | 0.337 | 1.000 |  |
| **MDD-W** | -0.069 | -0.023 | 0.247 | 0.409 | 1.000 |
