# Supplementary material for: Strengths and limitations of computer assisted telephone interviews (CATI) for nutrition data collection in rural Kenya
Source: PLoS One. 2019 Jan 30;14(1):e0210050. doi: 10.1371/journal.pone.0210050 (PMC6353544; doi:10.1371/journal.pone.0210050)
Supplement: S11 Table — (DOCX) [file pone.0210050.s011.docx]

**S11 Table. Correlations among MAD components and demographic indicators.**

|  | **Age** | **HH Size** | **Phones** | **PPI** | **MDD** | **Meals** |
| --- | --- | --- | --- | --- | --- | --- |
| **Age** | 1.000 |  |  |  |  |  |
| **HH Size** | 0.258 | 1.000 |  |  |  |  |
| **Phones** | 0.041 | 0.161 | 1.000 |  |  |  |
| **PPI** | -0.139 | -0.544 | 0.280 | 1.000 |  |  |
| **MDD** | 0.008 | -0.118 | 0.147 | 0.296 | 1.000 |  |
| **Meals** | -0.034 | -0.071 | 0.133 | 0.240 | 0.391 | 1.000 |
